# Supplementary material for: WordCommentsAnalyzer: A windows software tool for qualitative research
Source: F1000Res. 2018 Sep 4;7:536. Originally published 2018 May 3. [Version 2] doi: 10.12688/f1000research.14819.2 (PMC6137411; doi:10.12688/f1000research.14819.2)
Supplement: Supplementary file 3 [file f1000research-7-17614-s0002.tgz › b2a79496-4ada-47c4-9682-a99ce867836d.zip!/Eight Iranian Health Journals/Yafteh _38.docx]

© Yafteh  . This work is licensed under CC BY 4.0 (https://creativecommons.org/licenses/by/4.0/).

Volume & Issue: دوره 11 - شماره 3

Title: ارتباط بین اختلالات الکترولیتی، میزان ESR، CRP و پلاکت با شدت بیماری کاوازاکی

http://yafte.lums.ac.ir/article-1-159-fa.html

بیماری کاوازاکی یک واسکولیت حاد تب دار دوران کودکی است که در سراسر دنیا روی می دهد و در این بین آسیایی ها بالاترین خطر ابتلا را دارند. تقریباً 20% بیماران درمان نشده، دچار آنوریسم شریان کرونر، انفارکتوس میوکارد و مرگ ناگهانی می شوند. بیماری کاوازاکی علت اصلی ایجاد بیماری اکتسابی قلبی در کودکان ژاپنی و آمریکایی است. هدف از انجام این مطالعه، تعیین شدت بیماری کاوازاکی در کودکان بر اساس یافته های آزمایشگاهی و اکوکاردیوگرافیک می باشد. مواد و روش ها: در این مطالعه مقطعی، کلیه بیمارانی (61n=) که از 1/10/1383 تا 1/11/1386 با تشخیص اولیه بیماری کاوازاکی در بیمارستان کودکان مفید بستری شدند، بررسی شده و پس از حذف بیمارانی که معیارهای خروج از مطالعه را داشتند، 50 بیمار در دو گروه شدید (24n=) و خفیف (26n=) وارد مطالعه شدند. برای کلیه کودکان اکوکاردیوگرافی رنگی در زمان تشخیص، 3-2 هفته و 8-6 هفته پس از شروع بیماری انجام شد. تمامی موارد اکوکاردیوگرافی رنگی، فقط توسط یک نفر فوق تخصص قلب و عروق انجام شد که از ویژگیهای کودکان مطلع نبود. تمام بیماران تا 12 هفته پس از ترخیص پیگیری شدند. یافته ها: از 50 بیمار مورد مطالعه، 32 نفر (64 %) پسر و 18 نفر (36 %) دختر و نسبت پسر به دختر 1 : 8/1 بود. 100% بیماران تب، 82% تغییرات مخاط و لبها، 78% کونژونکتیویت دوطرفه غیر چرکی، 64% پوسته ریزی اندام ها، 58% راش و 40% آدنوپاتی گردنی داشتند. آنوریسم عروق کرونر در 2% بیماران دیده شد. در هیچیک از کودکان مبتلا، موردی از عود بیماری کاوازاکی مشاهده نگردید. بر اساس یافته های آزمایشگاهی و اکوکاردیوگرافیک، بیماران به 2 گروه با بیماری کاوازاکی شدید (24n=) و خفیف (26n=) تفکیک شدند. از نظر میانگین سنی، میانگین زمان بستری و بروز راش، بین دو گروه، اختلاف آماری معنی داری مشاهده شد. از نظر پتاسیم کمتر از 4/3 میلی اکی والان در لیتر، بین دو گروه، اختلاف آماری معنی داری مشاهده نشد. از نظر سدیم کمتر از 135 میلی اکی والان در لیتر، بین دو گروه، اختلاف آماری معنی داری مشاهده شد (01/0p=) . از نظر شمارش پلاکتها، سرعت سدیمانتاسیون اریتروسیتها و موارد CRP ≥ +++ ؛ بین دو گروه، اختلاف آماری معنی داری مشاهده نشد. از نظر بروز نارسایی دریچه میترال و نیز دریافت 2 نوبت IVIG، بین دو گروه، اختلاف آماری معنی داری مشاهده شد (به ترتیب 02/0p= و 008/0p=) . بحث و نتیجه گیری: در این مطالعه ما نشان دادیم که با زودتر انجام دادن اکوکاردیوگرافی داپلر رنگی و اندازه‌گیری CRP، ESR و سدیم می‌توان فرم شدید بیماری کاوازاکی را سریعتر و آسان تر تشخیص داد.

Title: مقایسه واکنش پاسخ به استرس در دو روش بیهوشی عمومی و بیحسی نخاعی در اعمال جراحی ژنیکولوژیک

http://yafte.lums.ac.ir/article-1-160-fa.html

مقدمه: واکنش پاسخ به استرس به دنبال جراحی و بیهوشی شامل طیف وسیعی از تغییرات هماتولوژیک و ایمییونولوژیک و اندوکرینولوژیک است که قسمتی از این تغییرات شناخته شده اند. در این مطالعه جهت بررسی و مقایسه اثر دو روش بیهوشی عمومی و بیحسی نخاعی روی واکنش پاسخ به استرس تعداد کل و رده های مختلف گلبول های سفید و سطح سرمی کورتیزول و پروتئین (CRP) C _ reactive به عنوان شاخصهایی از واکنش پاسخ به استرس مورد بررسی قرار گرفتند. مواد و روش ها: مطالعه حاضر بصورت کارآزمایی بالینی روی خانمهای 45- 25 ساله کاندید عمل جراحی ترمیم رکتوسل- سیستول درASA CLASS 1 و حاضر به همکاری در این تحقیق در بیمارستان عسلی خرم آباد وابسته به دانشگاه علوم پزشکی لرستان در سال84 انجام گرفت. بیماران به صورت تصادفی به دو گروه بیهوشی عمومی(GA)(29نفر) و بی حسی نخاعی (SA) (27نفر) در سطح T 10 تقسیم شدند. قبل از عمل و 12 ساعت بعد از عمل نمونه خون بیماران جهت بررسی تغییرات تعداد کل و رده های مختلف گلبول های سفید و سطح سرمی کورتیزول و CRP به آزمایشگاه فرستاده شد. یافته ها: با وجود عدم تفاوت واضح میانگین تعداد گلبولهای سفیددر قبل از عمل بین دو گروه میانگین تعداد گلبول های سفید در بعد از عمل بطور معنی داری در گروه بیهوشی عمومی نسبت به گروه بیحسی نخاعی بالاتر بود. در درصد پلی مورفو نو کلیرها و لنفوسیت وایوزینوفیل و منوسیت چه قبل وچه بعد از عمل اختلاف معنی داری بین دو گروه وجود نداشت. با وجود پایین بودن میانگین سطح سرمی کورتیزول قبل از عمل در گروه بیهوشی عمومی در 12 ساعت بعد سطح کورتیزول در این گروه نسبت به گروه بیحسی نخاعی بطور واضحی بالاتر بود. CPR در زمان قبل از عمل و 12 ساعت بعد از عمل در دو گروه تفاوتی نداشت. بحث و نتیجه گیری: بر اساس این نتایج بنظر میرسد که بیحسی نخاعی در سطح T 10 واکنش به استرس را مهار کرده و سبب کاهش کورتیزول و تعداد گلبول های سفید در بعد از عمل می شود و تأثیر آن احتمالاً از طریق مهار عبور امواج عصبی در سطح نخاع است.

Title: تأثیر روغن بذر گیاه گاو زبان بر پسوریازیس ولگاریس

http://yafte.lums.ac.ir/article-1-205-fa.html

پسوریازیس ولگاریس یک بیماری شایع، مزمن و عود کننده پوست است که در هر سن و جنسی دیده می شود. شیوع آن در جامعه 5/1 تا 3 درصد و علت بیماری نامشخص است. ضایعات بیماری به شکل پاپول پوسته ای و خارش دار روی زمینه اریتماتوز بوده و در نواحی سر، سطوح اکستانسور، کف دست و پا و ناخن بیشتر دیده می شود. درمان روتین بیماری، تجویز کرتیکواستروئید موضعی و سیستمیک است. بررسی ها نشان داده اند که این بیماران دچار کمبود اسیدهای چرب آزاد بوده و در صورت رفع این کمبود ، علائم بیماری کاهش و حتی بهبود می یابد. گیاه گاو زبان از منابع غنی گامالینولینک اسید است که تأثیر آن در بهبود بیماریهای مزمن التهابی ثابت شده است. این بررسی به منظور تعیین اثر روغن بذر گاو زبان بر ضایعات پسوریازیس انجام شد

Title: مقایسه تاثیر رژیم غذایی کاهش وزن پرپروتئین – کم چربی با رژیم استاندارد در کنترل وزن

http://yafte.lums.ac.ir/article-1-161-fa.html

چاقی یکی از مهمترین مشکلات تغذیه ای دنیاست. رژیمهای کم کالری مناسب بهترین راه برای کاهش وزن محسوب می شود. هدف از این مطالعه مقایسه تاثیر دو نوع رژیم غذایی محدودیت انرژی در کاهش وزن بود مواد و روش ها: 76زن 55-22 ساله سالم به طور تصادفی در دو گروه : 37 نفردریافت کننده رژیم استاندارد (55% کربوهیدرات، 15% پروتئین، 30% چربی) و 39 نفر دریافت کننده رژیم پرپروتئین- کم چربی (55% کربوهیدرات، 25% پروتئین و 20% چربی) به مدت 3 ماه مورد مطالعه قرار گرفتند. میزان محدودیت کالری در هر دو گروه 1000 کالری کمتر از نیاز روزانه بود. در پایان هر ماه بیماران ویزیت گردیده و انرژی رژیم غذایی تعدیل می گردید. متغیرهای نمایه توده بدنی، دور کمر، دور باسن و وزن در ابتدا و انتهای مطالعه محاسبه و اندازه گیری شد. یافته ها: مقدار BMI در گروه استاندارد و پرپروتئین به ترتیب :96/0±43/4 و 76/0±15/4 کیلوگرم بر متر مربع کاهش داشت. میزان کاهش وزن در گروه استاندارد 04/2 89/10 و در گروه پرپروتئین 73/1 48/10 بود. نسبت دور کمر به باسن در دو گروه استاندارد و پرپروتئین به ترتیب 014/0±020/0 و 014/0±018/0 کاهش داشت. این تفاوتها بین دو گروه معنی دار نبود. بحث و نتیجه گیری: هر دو نوع رژیم غذایی در کاهش نمایه توده بدنی، وزن، دور کمر، دور باسن و نسبت دور کمر به باسن موثر بودند. بنابراین می توان از رژیم پرپروتئین –کم چربی نیز بعنوان جایگزین رژیم استاندارد استفاده نمود.

Title: بررسی شیوع انواع اختلالات خواب در کودکان دبستانی 7-12 ساله تحت مطالعه در شهر خرم آباد در سال تحصیلی 86-85

http://yafte.lums.ac.ir/article-1-162-fa.html

از آنجایی که جدی ترین عارضه مربوط به اختلالات خواب کودکان ، مشکلات شناختی است و این مشکل کودکان را در معرض اختلالات تحصیلی، مشکلات خانوادگی و اجتماعی و روانی قرار می دهد، مطالعه انواع اختلالات خواب و نحوه مقابله با آنها مهم است. مطالعه حاضر به منظور بررسی شیوع انواع اختلالات خواب در کودکان دبستانی 7-12 ساله شهر خرم آباد در سال تحصیلی 86-85 انجام گرفته است. مواد و روش ها: ر این مطالعه توصیفی مقطعی 364 نفر از دانش آموزان مقطع ابتدایی شهر خرم آباد به روش تصادفی چند مرحله ای در هر دو جنس پسر و دختر به تعداد مساوی انتخاب و بررسی شدند. روش جمع آوری اطلاعات پرسشنامه بود. یافته ها: نتایج شیوع انواع اختلالات خواب را به ترتیب شیوع به این صورت نشان داد: تنفس با دهان باز 7/35 درصد ، صحبت کردن در خواب 7/24 درصد، خروپف عادتی 3/20 درصد، کابوس شبانه 8/19 درصد، دندان قروچه 9/15 درصد ، خواب آلودگی مفرط روزانه 10 درصد، شب ادراری ثانویه 2/8 درصد، شب ادراری اولیه 1/7 درصد، آپنه خواب 6/6 درصد، راه رفتن در خواب 6/6 درصد . همچنین آزمون های آماری نشان داد میان شب ادراری اولیه و ثانویه با جنس رابطه معناداری برقرار بوده و در جنس پسر شیوع بیشتری داشت. (004/0= P-value) همچنین میان دندان قروچه و خروپف ، آپنه و خروپف، تنفس با دهان باز و خروپف ارتباط معناداری برقرار بود. (001/0> P-value). بحث و نتیجه گیری: با توجه به یافته های تحقیق ، تنفس با دهان باز شایعترین اختلال خواب در جامعه مورد مطالعه بود که ارتباط نزدیک و معناداری با خروپف شبانه داشت که لزوم بررسی از نظر مشکلات طبی و جسمی زمینه ای و روانی را مطرح می سازد. لذا پیشنهاد می شود که ، به دلیل قابلیت درمان محافظه کارانه و غیر دارویی در اغلب اختلالات خواب و سودمند بودن این روش در نتایج مطالعه، بالا بردن سطح آگاهی خانواده ها از طریق مدارس و جلسات اولیاء و مربیان و کاهش شرایط استرس آور، انجام شود.

Title: جداسازی و بررسی پتانسیل بیولوژیکی باکتریهای تجزیه کننده فنانترن

http://yafte.lums.ac.ir/article-1-163-fa.html

هیدروکربن های آروماتیک چند حلقه ای از مواد شیمیایی بالقوه خطرناک برای محیط زیست بوده و منجر به نگرانی های بهداشتی شوند. این ترکیبات به علت دارا بودن خصوصیاتی همچون سمیت، جهش زایی و سرطان زایی توسطUSEPA در ردیف آلاینده های مقدم قرار گرفته اند. هیدروکربن های آروماتیک چند حلقه ای به سختی تجزیه پذیر است. کاربرد فرایند اصلاح زیستی (Bioremediation) برای حذف هیدروکربن های آروماتیک چند حلقه ای از خاک های آلوده یکی از گزینه های اقتصادی و مطلوب می باشد. مواد و روش ها: در این مطالعه فنانترن (ترکیب سه حلقه ای از ترکیبات هیدروکربن های آروماتیک چند حلقه ای) به عنوان نماینده این ترکیبات انتخاب گردید و در دو دامنه غلظت (mg/kg100 و mg/kg500) مورد مطالعه قرار گرفت. در ابتدا میکروارگانیسم های تجزیه کننده هیدروکربن های حلقوی جداسازی و پس از سازگاری وغنی سازی، باکتریها شناسایی شدند. آنالیز باکتری به روش تست تأییدی و کیت api انجام گرفت وچهار باکتری سودوموناس spp، باسیلوس، سودوموناس آئروژنوزا و اسینتوباکتر تشخیص داده شد. فنانترن موجود در خاک با استفاده از روش التراسونیک استخراج شد و جهت سنجش میزان فنانترن از دستگاهHPLC استفاده گردید. یافته ها: بررسی مطالعات اصلاح زیستی در خاکهای آلوده به فنانترن نشان داد که حذف موثر فنانترن توسط باکتری سودوموناس بیشتر از باکتری های دیگر است همچنین حذف موثر در 45 روز اول اتفاق می افتد و سپس میزان حذف کاهش می یابد. از یافته های دیگر در این مطالعه این بود که غلظت پایین فنانترن سریعتر از غلظت بالا حذف می شود و همچنین مقایسه تجزیه زیستی فنانترن توسط گونه های خالص و کنسرسیوم نشان داد که سرعت حذف فنانترن توسط کنسرسیوم در ابتدای فرایند از باکتری های دیگر بیشتر است. بحث و نتیجه گیری: نتایج حاصل نشان داد باکتریهای جداسازی شده از محیط کشت غنی شده به جز سودوموناس که تنها توانایی تجزیه نفتالین را دارد، توانایی تجزیه هیدروکربنهای آروماتیک چند حلقه ای را از خود نشان دادند. جمعیت باکتریها در طول هفته اول کاهش و سپس رو به افزایش گذاشت. کاهش اولیه می تواند به دلیل مواجهه با فنانترن باشد اما باکتریها از محیط هایی که تنها منبع کربن آنها فنانترن بوده توانایی تکثیر مجدد را دارند.

Title: بررسی ارتباط بین نسبت دور کمر به دور باسن و میزان فشار خون در افراد مراجعه کننده به مراکز بهداشتی درمانی شهر اراک در سال 1387

http://yafte.lums.ac.ir/article-1-164-fa.html

شیوع چاقی در دنیا به طور قابل ملاحظه ای رو به افزایش است، به طوری که در جوامع علمی دنیا به عنوان یک اپیدمی مطرح گردیده است. چاقی می تواند سبب افزایش خطر ابتلا به بیماری های قلبی، دیابت، فشار خون بالا و برخی سرطان ها گردد. دور کمر و دور باسن نحوه توزیع چربی دربدن را نشان می دهند و تحت تاثیر عوامل مربوط به شیوه زندگی (مصرف سیگار، مصرف الکل و فعالیت فیزیکی) می باشند، و امروزه متداول ترین شاخص مورد استفاده برای شناسایی نحوه توزیع چربی در بدن نسبت دور کمر به دور باسن می باشد. این مطالعه به منظور بررسی ارتباط نسبت دور کمر به دور باسن با فشار خون در افراد مراجعه کننده به مراکز بهداشتی درمانی شهر اراک انجام شد. مواد و روش ها: در این مطالعه مقطعی و بر پایه جمعیت، 340 فرد 18 سال به بالا که طی سال 1387 به مراکز بهداشتی درمانی شهر اراک مراجعه نموده بودند، به طور تصادفی انتخاب شدند. اطلاعات با استفاده از پرسشنامه ای که حاوی اطلاعات دموگرافیک آنان بود جمع آوری گردید. دور کمر، دور باسن و فشار خون افراد مورد بررسی به روش استاندارد اندازه گیری شد. سپس نسبت دور کمر به دور باسن در کلیه افراد محاسبه گردید. داده ها با استفاده از نرم افزار آماری SPSS مورد تجزیه و تحلیل قرار گرفتند. برای توصیف متغیرها از آمار توصیفی نظیر جداول توزیع فراوانی، نسبتها، درصدها، میانگین، انحراف معیار و دامنه تغییرات استفاده شد. سپس جهت ارتباط سنجی‌ها از ضریب همبستگی پیرسون و ضرایب همبستگی جزئی استفاده ‌گردید. در ضمن جهت پیشگویی فشار خون بالا با استفاده از شاخص نسبت دور کمر به دور باسن از منحنی ROC استفاده شد. یافته ها: 6/32 درصد افراد تحت بررسی مرد و 4/67 درصد زن بودند. 2/16 درصد از مردان و 2/12 از زنان فشار خون بالا داشتند. نسبت دور کمر به دور باسن همبستگی معنی دار مستقیم با فشار خون داشت. سطح زیر منحنی ROC به ازای زنان 802/0 و به ازای مردان 815/0 محاسبه شد و بهترین مقدار بحرانی WHR جهت پیشگویی ابتلا به فشار خون در مردان و زنان به ترتیب 895/0 و 830/0 به دست آمد بحث و نتیجه گیری: یافته ها نشان داد که در جامعه افراد اراکی، WHRشاخص بهتری نسبت به دور کمر و دور باسن به تنهائی، برای پیشگویی فشار خون می باشد.

Title: بررسی وضعیت بایگانی پزشکی در بیمارستانهای آموزشی شهر خرم آباد در سال 1387

http://yafte.lums.ac.ir/article-1-167-fa.html

نتیجه اندیشه ها، فعالیت ها و اقدامات انجام شده در هر سازمان به صورت اسناد و مدارک حفظ ونگهداری می شوند؛ این سوابق که با صرف وقت و هزینه فراوان بوجود می آیند، حاوی اطلاعات وتجربیات گرانبهایی است که در پیشبرد هدف یا اهداف سازمان نقش مهمی بر عهده دارد. بنابراین می توان بایگانی مدارک پزشکی را حافظه سازمان نامید که ضعف در کارائی آن موجب خلل در کلیه فعالیت های بیمارستان می شود. بنابراین مطالعه حاضر با هدف بررسی وضعیت بایگانی در بیمارستانهای آموزشی شهر خرم آباد در سال 1387 انجام شد. مواد و روش ها: این پژوهش، مطالعه ای توصیفی- تحلیلی است که به صورت مقطعی انجام شده است. داده ها با استفاده از چک لیست و پرسشنامه های مبتنی بر استانداردهای واحد بایگانی پزشکی به روش مشاهده و مصاحبه گرد آوری گردید. چک لیست های تکمیل شده با استفاده از روش امتیاز بندی و آستانه ی تحلیل به کمک نرم افزارSpss مورد بررسی و تحلیل قرار گرفت. و برای ارزیابی کیفی واحد بایگانی پزشکی از پرسشنامه استفاده شد تا تحلیل و شفاف سازی وضعیت بایگانی با تمام جزئیات در هر یک از بیمارستانها صورت گیرد. یافته ها: درجه مطلوبیت موقعیت مکانی و فضا، پرسنل، روشهای ذخیره سازی و بازیابی پرونده های پزشکی، تجهیزات، دستورالعملها و رویه ها و سیستم مکانیزه واحد بایگانی پزشکی در بیمارستانهای مورد پژوهش با درصد امتیاز کلی(41%) در گروه متوسط قرار گرفت. بالاترین درصد تطابق با استاندارد مربوط به سیستم مکانیزه با(8/48%) و کمترین آن مربوط به موقعیت مکانی و فضا با (26%) بود. بحث و نتیجه گیری: در مجموع واحد بایگانی پزشکی در کلیه بیمارستانهای مورد مطالعه از وضعیت مطلوبی برخوردار نبود. بنابراین استفاده از فناوری های مدرن ذخیره سازی و بازیابی مدارک پزشکی مانند ایجاد بایگانی پزشکی الکترونیکی، اسکن نمودن مدارک پزشکی و ذخیره سازی دیجیتالی آنها با قابلیت بازیابی سریع، موجب ارتقا بازدهی و عملکرد این واحد، استفاده بهینه از منابع، صرفه جویی هزینه و ارتقا مراقبت از بیمار می شود.

Title: بررسی و مقایسه ‌آموزش فراشناختی پردازش هیجانی توام با دارودرمانی با درمان استاندارد (دارودرمانی ) در بهبود مهارت های عاطفی، شناختی و اجتماعی بیماران دوقطبی

http://yafte.lums.ac.ir/article-1-168-fa.html

درمان های روان شناختی اختلال دو قطبی به مقدار زیادی مورد غفلت قرار گرفته است. هدف از این پژوهش تعیین اثربخشی آموزش فراشناختی پردازش هیجانی همراه با درمان دارویی در مقایسه با درمان استاندارد در افزایش مهارت های عاطفی، شناختی و اجتماعی(MEPT) بیماران مبتلا به اختلال دو قطبی است. مواد و روش ها: پژوهش از نوع نیمه تجربی با گروه کنترل بوده و نمونه شامل 32 زن (40-16) که بر اساس ملاکهای تشخیصی DSM-IV-R تشخیص اختلال دو قطبی نوع اول گرفتند ، بود. که به طور تصادفی از بین مراجعان بیمارستان ها و کلینیک های روانپزشکی شهر اصفهان انتخاب شده اند. یکی از گروهها تنها دارو دریافت کرد و به عنوان گروه کنترل در نظر گرفته شد و گروه دیگر آموزش فراشناختی پردازش هیجانی توام با دارو دریافت کرد. ابزارهای مورد استفاده در این پژوهش، مصاحبه نیمه ساختاری براساس معیارهای DSM-IV-R و پرسشنامه مانیا ( Bech و همکاران، 1979) و مقیاس 17 آیتمی افسردگی (Hamilton ، 1980) برای استفاده مانیا و افسردگی استفاده شده است. برای اندازه گیری برخی مهارتهای عاطفی از برخی زیرمقیاس های هوش هیجانی Cooper) و همکاران، 1999) و درمورد مهارت های شناختی از پرسشنامه و نگرش های بدکنشی ( Power و همکاران، 1994) ، خودکنترلی( Rosenbaum ، 1980) و بینش ( David و همکاران، 1992) استفاده شد. برای اندازه گیری عملکرد اجتماعی از پرسشنامه عملکرد اجتماعی ( Hurry و همکاران، 1983)، استفاده گردید. برای کنترل اثرمتغیر وضعیت اقتصادی ، اجتماعی، فرهنگی آزمودنی ها بر متغیر مستقل از پرسشنامه محقق ساخته استفاده شد. یافته ها: شیوه (MEPT) برافزایش تمام زیرمقیاس های نمره کل مهارت های عاطفی موثر بوده (05/0 P<) و در مورد مهارت های شناختی این شیوه درکاهش افکار ناکارآمد (000/0= P) و افزایش بینش (05/0 P<) و خودکنترلی (000/0= P) موثر بوده است. در مورد مهارت های اجتماعی این شیوه در افزایش مهارت های اجتماعی اثربخش بوده است(02/0 = P). بحث و نتیجه گیری: استفاده از شیوه آموزشی فراشناختی پردازش هیجانی به انضمام شیوه درمان دارویی اثربخشی درمان را افزایش می دهد. بنابراین لازم است که درمانگران در این زمینه آموزش ببینند.

Title: بررسی موانع انجام پژوهش از دیدگاه اعضای هیات علمی دانشگاه علوم پزشکی لرستان

http://yafte.lums.ac.ir/article-1-169-fa.html

پژوهش روندی است فعالانه و سامان مند برای کشف ، تعبیر و بازنگری پدیده ها ، رخدادها وفرضیه هاست. اولین گام برای سامان بخشیدن به امر پژوهش در جامعه، دستیابی به درک درستی از توانمندی های امکانات موجود و نیز پی بردن به نقاط ضعف و قوت برنامه های تحقیقاتی است. پژوهش حاضر با منظور تعیین موانع انجام پژوهش از دیدگاه اعضای هیأت علمی دانشگاه علوم پزشکی لرستان انجام شد. مواد و روش ها: این مطالعه توصیفی مقطعی به منظور تعیین موانع پژوهش از دیدگاه اعضاء هیات علمی دانشگاه علوم پزشکی لرستان انجام گرفت. نمونه پژوهش شامل تمامی اعضاء هیات علمی شاغل در دانشگاه علوم پزشکی لرستان بجز دانشکده پرستاری الیگودرز بودند. ابزار گردآوری داده ها توسط پرسشنامه ای چهار قسمتی بر اساس مقیاس چهار گزینه ای لیکرت صورت گرفت اطلاعات 93 پرسشنامه توسط آزمونهای t مستقل، آنالیز واریانس مورد بررسی و تجزیه و تحلیل قرار گرفتند. یافته ها: در رابطه با موانع شخصی، در مجموع، کمبود وقت به دلیل تدریس زیاد (2/32 درصد) و مسؤلیتهای اجتماعی و اجرائی (4/21 درصد) بیشترین و عدم علاقه شخصی به امور پژوهش (7/60 درصد) و نداشتن تبحر کافی در نوشتن مقاله و تدوین پروپوزال (6/50 درصد) کمترین موانع شخصی بودند. در خصوص موانع سازمانی سختگیری در تصویب طرحهای مورد علاقه پزوهشگر و مقررات دست و پاگیر اداری . با (2/49 درصد) و پروسه طولانی تصویب طرحهای پژوهشی و کافی نبودن امتیازات پژوهشی جهت ارتقاء پژوهشگران با (1/39 درصد) بیشترین مانع سازمانی و مشکلات مربوط به استخراج آماری نتایج و محدودیتهای اخلاقی در انجام پژوهش (9/34 درصد) و اجبار در استفاده پژوهشگر از یک روش و چارچوب خاص (9/27 درصد) کمترین موانع سازمانی گزارش گردید. در ضمن بین میانگین نمرات موانع شخصی در بین دانشکده ها اختلاف معنی داری وجود نداشت در صورتیکه بین نمرات موانع سازمانی اختلاف معنی دار بود. بحث و نتیجه گیری: با توجه به نقش محوری دانش و پژوهش که از مهمترین مشخصه های نگاه به آینده است دولتها و سازمانها و ملتها باید تغییر و آینده را بهتر و بیشتر درک نماید. چرا که ما همگان در آینده ای زندگی و کار خواهیم نمود که متفاوت از امروز خواهد بود. با توجه به نتایج بدست آمده برطرف کردن موانع اساسی ذکر شده .جهت رسیدن به آینده پژوهشی پربارتر ضروری به نظر می رسد.

Title: بررسی عوامل مؤثر در مصرف داروهای ممنوعه و مکملهای نیروزا در دانش آموزان دبیرستانی

http://yafte.lums.ac.ir/article-1-170-fa.html

مصرف مواد مختلف نیروزا جهت بهبود عملکرد ورزشی یک مشکل بزرگ در سراسردنیا محسوب می شود. هدف از انجام این تحقیق تعیین میزان شیوع مصرف مواد نیروزا و آگاهی دانش آموزان استان لرستان درمورد دوپینگ و مصرف مواد نیروزا بوده است مواد و روش ها: این تحقیق با استفاده از یک پرسشنامه ی خودگزارش دهی و محقق ساخته بر روی 1180 دانش آموزان پسر و دختر در 8 شهر و یک بخش (طرحان از توابع شهرستان کوهدشت) انجام گرفت. پس ازحذف پرسشنامه های ناقص، تعداد 992 پرسشنامه کدگذاری شده و توسط نرم افزار SPSS ویرایش شماره 15 بوسیله آمار توصیفی (میانگین ها، جداول متقاطع و توزیع فراوانی) و آمار استنباطی (آزمونهای تحلیل واریانس، تحلیل رگرسیون، کای دو، تحلیل عاملی، آلفای کرونباخ و ضریب همبستگی) مورد تجزیه و تحلیل قرارگرفتند. یافته ها: نتایج این تحقیق نشان داد که 92 نفر (3/9%) از دانش آموزان شرکت کننده در این تحقیق اعتراف به مصرف حداقل یک ماده نیروزا نمودند، که 37 نفر (7/3%) از این دانش آموزان از داروهای ممنوعه و دوپینگی و 71 نفر (1/7%) از دانش آموزان نیز از مکمل های ورزشی استفاده کرده بودند (البته برخی افراد هم دارو و هم مکمل مصرف کرده بودند). البته میزان شیوع مواد نیروزا در بین دانش آموزان پسر برابر با 4/13% و در بین دانش آموزان دختر برابر با 3/4% بود. تحلیل اطلاعات در این تحقیق نشان داد که عوامل موثر در شیوع مصرف مواد نیروزا شامل سن، جنس، قد، وزن، محل سکونت، میزان درآمد خانواده، ورزشکار بودن، رشته ی ورزشی، شیوه ی فعالیت ورزشی، داشتن سابقه ی قهرمانی، رشته ی ورزشی قهرمانی، سطح قهرمانی، نگرش به سمت دوپینگ، آگاهی از دوپینگ و شناخت نام مواد نیروزا می باشد (p<0. 05). همچنین مهمترین متغیرهای پیش بینی کننده ی مصرف مواد نیروزا به ترتیب شامل متغیرهای نگرش به سمت دوپینگ، وزن، شناخت نام مواد نیروزا، منطقه ی محل سکونت دانش آموز و رشته ی ورزشی که دانش آموز در آن فعالیت می کند می باشند که دارای ضریب همبستگی چند متغیره 0. 475+ R= با مصرف مواد نیروزا می باشندکه این میزان بیانگر 23% از تغییر پذیری مصرف مواد نیروزا می باشد. یعنی با کنترل این 5 متغیر می توان 23% مصرف مواد نیروزا را کنترل نمود. بحث و نتیجه گیری: نتایج این تحقیق نشان داد که دوپینگ و مصرف مواد نیروزا در بین دانش آموزان دبیرستانی یک واقعیت انکار ناپذیر بوده و در صورت عدم توجه مسئولین و برنامه ریزان در این مورد، هر روز شاهد آسیب های بیشتر جوانان و نوجوانان عزیز میهن اسلامی مان خواهیم بود.

Title: ارزیابی مقایسه ای میزانهای بقاء در مبتلایان به سرطان های معده و مری

http://yafte.lums.ac.ir/article-1-171-fa.html

در ایران، سرطان ها به عنوان دومین علت مرگ و میر و بطور خاص سرطان های معده و مری به عنوان یکی از کشنده ترین بیماریهای سرطانی مطرح می باشند. مطالعه حاضر با هدف بررسی و مقایسه میزان های بقاء در مبتلایان به سرطان های معده و مری صورت پذیرفت. مواد و روش ها: پس از استخراج اطلاعات کلیه بیماران مبتلا به سرطان های معده و مری ثبت شده در مرکز ثبت تومور استان فارس، پیگیری وضعیت بقاء ایشان به صورت تلفنی انجام شد. آنالیز اطلاعات با استفاده از دو روش آماری کاپلان- میر و جدول عمر صورت پذیرفت. یافته ها: در مجموع 442 بیمار مبتلا به سرطان معده و 161 مورد مبتلا به سرطان مری مورد بررسی قرار گرفتند. میزان های بقاء کلی 1 و 5 ساله در مبتلایان به سرطان معده و مری در روش کاپلان میر به ترتیب 54 و 16 درصد و 53 و9 درصد بدست آمد. همچنین این میزان ها توسط روش جدول عمر 57 و 16 درصد در سرطان معده و 58 و10 درصد حاصل شد. که در هر دو روش اختلاف معنی داری میان میزان های بقا در دو سرطان مشاهده نشد. بحث و نتیجه گیری: نتایج نشان داد که علیرغم برخی گزارشات، بین میزان های بقا در دو سرطان اختلاف آماری معنی داری وجود ندارد و تنها تعداد کمی از بیماران 5 سال پس از تشخیص بیماریشان زنده می مانند. لذا لزوم انجام اقداماتی در زمینه بالا بردن طول عمر و کیفیت زندگی این بیماران ضروری بنظر می رسد.

Title: بررسی کیفیت زندگی و ابعاد آن در بیماران دیابتی مراجعه کننده به درمانگاه بیمارستان شهداء شهر خرم آباد (در سال 1387 )

http://yafte.lums.ac.ir/article-1-173-fa.html

بیماریهای مزمنی همچون دیابت از بیماری های غیر قابل درمانی است که می توان آن را کنترل کرد، اما بیمار همواره در معرض عوامل تنش زای جسمی، روانی و اجتماعی قرار دارد، لذا در این مطالعه قصد بر آن است ضمن تعیین کیفیت زندگی بیماران دیابتی، ابعاد مختلف آن نیز سنجیده شود. مواد و روش ها: این پژوهش، یک مطالعه توصیفی است که در سال 1387 انجام شد، جامعه آماری، کلیه بیماران مبتلا به دیابت در شهر خرم آباد بودند که جهت پیگیری درمان بیماری خود به بیمارستان مراجعه می‌کردند که 150 نفر از آنان با استفاده از روش تصادفی ساده انتخاب و به پرسشنامه مشتمل بر دو بخش مشخصات دموگرافیک و کیفیت زندگی به صورت پرسشگری و خودایفا پاسخ دادند. کیفیت زندگی از سه قسمت تشکیل شده است: بعد جسمی، بعد روانی و بعد اجتماعی. برای اعتبار پرسشنامه از روش اعتبار محتوا از نوع اعتبار صوری استفاده شد و برای تعیین پایایی از ضریب آلفای کرونباخ استفاده شد، میزان کیفیت زندگی بیماران بر اساس نمره به دست آمده به دو قسمت نا مطلوب (نمرات کمتر از 54) و مطلوب (نمرات مساوی یا بیشتر از 54) تعریف شد. یافته ها: نتایج نشان داد کیفیت زندگی 3/58 درصد آزمودنی ها نامطلوب و 7/41 درصد مطلوب بوده است، ضمن اینکه 9/79 درصد در بعد جسمی، 5/71 درصد در بعد روانی دارای کیفیت زندگی نامطلوب هستند، در حالیکه 6/80 درصد در بعد اجتماعی از کیفیت زندگی مطلوب برخوردار بودند، همینطور متغیرهای سن، جنس، وضعیت تأهل، وضعیت شغلی، وضعیت تحصیلی و مدت ابتلا به بیماری دیابت از نظر آماری دارای ارتباط معنی دار با کیفیت زندگی می باشد. بحث و نتیجه گیری: با توجه به اینکه نیمی از بیماران در ابعاد جسمی و روانی دارای کیفیت زندگی نامطلوب بودند توصیه می شود، مسئولین برنامه هایی با محتوای مشاوره ای، آموزشی و مادی اتخاذ نمایند که در ارتقای کیفیت زندگی بیماران تأثیر گذار باشد.
